# Up-regulation of LCN2 in the anterior cingulate cortex contributes to neural injury-induced chronic pain

**DOI:** 10.3389/fncel.2023.1140769

**Published:** 2023-06-08

**Authors:** Xiang-Jie Song, Chen-Ling Yang, Danyang Chen, Yumeng Yang, Yu Mao, Peng Cao, Aijun Jiang, Wei Wang, Zhi Zhang, Wenjuan Tao

**Affiliations:** ^1^Hefei National Research Center for Physical Sciences at the Microscale, Division of Life Sciences and Medicine, University of Science and Technology of China, Hefei, China; ^2^Key Laboratory of Oral Diseases Research of Anhui Province, College and Hospital of Stomatology, Anhui Medical University, Hefei, China; ^3^Department of Physiology, School of Basic Medical Sciences, Anhui Medical University, Hefei, China; ^4^Department of Neurology, Stroke Center, The First Affiliated Hospital of University of Science and Technology of China, Division of Life Sciences and Medicine, University of Science and Technology of China, Hefei, China; ^5^Department of Endocrinology and Laboratory for Diabetes, The First Affiliated Hospital of University of Science and Technology of China, Division of Life Sciences and Medicine, University of Science and Technology of China, Hefei, China

**Keywords:** chronic pain, ACC, LCN2, chemogenetics, neuronal hyperactivity, *in vivo* 2P calcium imaging

## Abstract

Chronic pain caused by disease or injury affects more than 30% of the general population. The molecular and cellular mechanisms underpinning the development of chronic pain remain unclear, resulting in scant effective treatments. Here, we combined electrophysiological recording, *in vivo* two-photon (2P) calcium imaging, fiber photometry, Western blotting, and chemogenetic methods to define a role for the secreted pro-inflammatory factor, Lipocalin-2 (LCN2), in chronic pain development in mice with spared nerve injury (SNI). We found that LCN2 expression was upregulated in the anterior cingulate cortex (ACC) at 14 days after SNI, resulting in hyperactivity of ACC glutamatergic neurons (ACC^Glu^) and pain sensitization. By contrast, suppressing LCN2 protein levels in the ACC with viral constructs or exogenous application of neutralizing antibodies leads to significant attenuation of chronic pain by preventing ACC^Glu^ neuronal hyperactivity in SNI 2W mice. In addition, administering purified recombinant LCN2 protein in the ACC could induce pain sensitization by inducing ACC^Glu^ neuronal hyperactivity in naïve mice. This study provides a mechanism by which LCN2-mediated hyperactivity of ACC^Glu^ neurons contributes to pain sensitization, and reveals a new potential target for treating chronic pain.

## Introduction

The development of chronic pain caused by disease or tissue injury is usually spontaneous, persistent, or severe, and induces an amplified pain response (Fayaz et al., 2016; Colloca et al., 2017; Cohen et al., 2021). At present, chronic pain remains extremely difficult to treat with conventional analgesics due to the complexity of its underlying mechanisms. That is, drugs not only have little effect, but are also accompanied by severe side effects (Kuo et al., 2015; Wongrakpanich et al., 2018; Micheli et al., 2020). It is thus both urgent and necessary to characterize the possible molecular and cellular mechanism(s) through which chronic pain develops.

Pathological neuronal plasticity is widely accepted to contribute to the central sensitization of pain (Luo et al., 2014; Ji et al., 2018; Wang et al., 2018; Treede et al., 2022; Zhou et al., 2022), which can be observed in many pain-associated regions, including the spinal cord (Bittar et al., 2017; Liu et al., 2017), anterior cingulate cortex (ACC) (Bliss et al., 2016; Meda et al., 2019), thalamus (Park et al., 2018; Zhu et al., 2021), amygdala (Corder et al., 2019; Zhou et al., 2019), and somatosensory cortex (Potter et al., 2016; Zhu et al., 2021). In particular, the ACC is known to participate in transmitting internal and external sensory stimuli and is known to be involved in the processing and conversion of pain information (Yoshino et al., 2010; Koga et al., 2015; Meda et al., 2019). Increased activity of ACC neurons has also been shown to contribute to inflammation- or injury-induced pain and emotion (Koga et al., 2010, 2015). However, the molecular mechanisms that drive this neuronal activity remain largely unknown.

Lipocalin-2 (LCN2), a 25-kDa secreted protein, also called 24p3 or neutrophil gelatinase associated lipid transporter (NGAL) (Kjeldsen et al., 1993; Flo et al., 2004), has been shown to play a crucial role in immune system diseases (Xu et al., 2015; Chen et al., 2020), metabolic diseases (Elkhidir et al., 2017; Moschen et al., 2017), central nervous system diseases (Ferreira et al., 2015; Jha et al., 2015; Suk, 2016), kidney damage (Viau et al., 2010; Ashraf et al., 2016), iron homeostasis (Xiao et al., 2016; Roemhild et al., 2021), and other diseases (Makhezer et al., 2019; Staurenghi et al., 2021). LCN2 is synthesized and secreted in response to inflammation, injury, or infection (Jha et al., 2015), and has been implicated in the regulation of a variety of biological/behavioral responses via its cell surface receptor (24p3R), including cognition and emotion by regulating neural plasticity (Ferreira et al., 2013; Jha et al., 2015). However, it is unknown whether and how LCN2 might regulate the neuronal plasticity that mediates chronic pain.

In this study, we investigated how LCN2 in the ACC is involved the development and maintenance of chronic pain. Using electrophysiological recording, *in vivo* two-photon (2P) calcium imaging, and fiber photometry, we found ACC^Glu^ neurons become hyperactive in the formation of pain sensitization following spared nerve injury (SNI). Chemogenetic inhibition of ACC^Glu^ neuronal activity resulted in alleviating chronic pain. In addition, we found that LCN2 expression levels were significantly higher in SNI 2W mice than in sham mice. Downregulating LCN2 expression by viral knockdown or by neutralizing antibody targeting LCN2 in the ACC led to inhibition of ACC^Glu^ neuronal activity accompanied by significant alleviation of mechanical nociceptive and thermal pain thresholds in SNI 2W mice. This study provides a theoretical basis for understanding the molecular and cellular mechanisms of chronic pain and suggests that LCN2 might serve as an effective target of therapeutic treatments of chronic pain.

## Materials and methods

### Animals

All the animal experiments were approved by the Animal Care and Use Committee of the University of Science and Technology of China. We used male mice aged 8–10 weeks for all experimental research, including C57BL/6J (Beijing Vital River Laboratory Animal Technology Co., Ltd., China), *CaMKII-Cre*, and *Ai14* (RCL-tdT) mice (Charles River or Jackson Laboratories, USA). Mice were group-housed five per cage. All mice had *ad libitum* access to food and water. They were housed at a stable temperature (23–25°C) with a 12-h light/dark cycle (lights on from 7:00 a.m. to 7:00 p.m.).

### Animal model of spared nerve injury

First, anesthesia was induced and maintained in all SNI mice by continuous inhalation of isoflurane using a gas anesthesia machine (3/2%). A small incision was made in the mouse’s left thigh’s skin. The muscle was gently and bluntly separated with a glass parting needle to expose the sciatic nerve bundle consisting of the gastrocnemius, common peroneal, and tibial nerves. Once the nerves are exposed, the common peroneal and tibial nerves are firmly ligated with non-absorbable 4-0 sutures and incised distally after these nerves are exposed to maintain the integrity of the nerves. The skin was then sutured and cleaned with iodine. A similar procedure was performed on the sham mice without damage to their nerves.

### Pain tests

Calibrated von Frey filaments were used for testing the mechanical withdrawal threshold of mice. To accustom them to the testing environment, mice were individually placed in a transparent plastic chamber on a wire mesh grid at least 30 min. Then we tested the withdrawal threshold of the planta using von Frey filaments on the middle of the plantar surface of the left hindpaws. The pressure of the von Frey filament was increased gradually. A positive response was considered when a mouse withdrew or licked its paw. The withdrawal threshold was tested every 10 min and the mean withdrawal threshold was calculated from three applications.

The Hargreaves test is used to assess the thermal pain threshold. The minimal delay in paw withdrawal after sensing a thermal stimulus is assessed three days after the mouse acclimatizes. Laser radiant heat (IITC, CA, USA) is provided to the left hind paw of mice. Thermal laser stimulation of the tested paw lasted only 20 s to prevent potential tissue damage. In this study, pain threshold is determined using the injured left hindpaws. The experimenters were blinded to group identity during the experiment and quantitative analyses.

### Open field test

The instrument used for open field test (OFT) was a white, single-sided, frosted acrylic chamber with dimensions of 50 cm × 50 cm × 60 cm and a central area of 25 cm × 25 cm. The mice were placed carefully in the central area to initiate the assay, and their movements were recorded for the first 6 min using video software. EthoVision XT 14 software (Noldus, Wageningen, Netherlands) was used to analyze the total distance traveled by the mice in the open field; total time spent in the central area, and the number of entries into the central area were recorded during the second 5 min of the experiment. After each mouse experiment, the open field box was cleaned with 75% ethanol and pure water to eliminate any interference due to smell.

### Elevated plus maze test

The elevated plus maze test (EPMT) consists of two open arms and two closed arms, each measuring 30 cm × 6 cm × 20 cm, which are crossed vertically to form a central area platform measuring 6 cm × 6 cm. The maze is situated 100 cm above the ground, and a dim light (∼20 lux) is used to acclimatize the mice. The mice were gently placed in the central area facing the open arms, and their movements were recorded for the first 6 min using behavioral recording software. The second 5 min of the experiment were analyzed using EthoVision XT 14 software (Noldus, Wageningen, Netherlands) to determine the total distance traveled by the mice and the number and time spent in the open arms. After each mouse experiment, the elevated plus maze apparatus was wiped with 75% ethanol and pure water to create a clean, odor-free experimental environment and eliminate any experimental errors due to odor.

### Immunofluorescence and imaging

Mice were first rapidly anesthetized with isoflurane (15 s), then deeply anesthetized by intraperitoneal injection of sodium pentobarbital and their hearts were sequentially infused with ice-cold saline (3 min) and 4% (w/v) paraformaldehyde (PFA) (4 min) via a peristaltic pump. Brains were fixed with 4% PFA for 6–8 h at 4°C and then dehydrated in 20 and 30% (w/v) sucrose until sunken. For immunofluorescence, 40 μm coronal sections were cut using a cryostat (Leica CM1860). Sections were incubated with blocking solution [PBS containing 0.3% (w/v) Triton X-100] for 1 h at room temperature and detected with primary antibodies, including anti-c-Fos (1:500, rabbit, Synaptic Systems), anti-glutamate (1:500, mouse, Sigma-Aldrich; 1:500, mouse, Sigma-Aldrich), anti-LCN2 (1:300, goat, BOSTER), anti-24p3R (1:500, rabbit, Sigma-Aldrich), anti-NeuN (1:500, mouse, Millipore), anti-Iba1 (1:500, rabbit, Wako), anti-GFAP (1:500, rabbit, Dako), and anti-GABA (1:500, mouse, Sigma-Aldrich) were incubated for 24 h at 4°C (Supplementary Table 2). The sections were washed with PBS three times, and incubated with the corresponding fluorophore-conjugated Alexa-Fluor 488 and Alexa-Fluor 594 secondary antibodies (Thermo Fisher) for 2 h at room temperature, then washed three times with PBS and localized with DAPI for nuclear staining of cells, and finally patched. Fluorescence signals were visualized using Zeiss LSM880 and LSM980 microscopes. We counted the expression of c-Fos, Glu, LCN2, 24p3R, NeuN, Iba1, GFAP, and GABA by ImageJ software; the slices randomly picked from per mouse were imaged and quantified for five mice per group. The mice used for immunofluorescence were pseudo-randomly assigned to the experimental group and the control group. Further analyses such as analysis of cell counts and colocalization were conducted using ImageJ software by an observer blind to condition.

### Hematoxylin and eosin staining

The mice were first perfused, and their brains were collected and fixed overnight in 4% PFA. After graded dehydration and hyalinization, the specimens were embedded in wax and sectioned (4 μm thick). The sections were then sequentially dewaxed in a gradient of water, xylene, and ethanol of different concentrations (100-95-80%). For hematoxylin and eosin (H&E) staining, paraffin sections were immersed in hematoxylin staining for 2–5 min, rinsed with tap water, fractionated with 1% hydrochloric acid for a few seconds, rinsed with tap water, returned to blue with 1% aqueous ammonia solution for 1 min, rinsed again with running water for a few seconds, then placed in eosin staining solution for a few seconds, and rinsed with running water. After H&E staining, the paraffin sections were dehydrated and transparent and then sealed with neutral gum. The histopathological changes of ACC were observed using an optical microscope (Olympus Optical Co. Ltd., Tokyo, Japan).

### Virus injection

Prior to surgery, a stereotactic frame (RWD, Shenzhen, China) was used to fix the mice under anesthesia by an intraperitoneal injection of pentobarbital (20 mg/kg). A heating pad was used to maintain the core body temperature of mice at 36°C. Using a calibrated glass microelectrode connected to an infusion pump, 150–250 nl of the virus was injected into the ACC (Micro 4, WPI, USA) at a rate of 50 nl/min, depending on the titer and expression of the virus (Supplementary Table 2). To stop virus leakage, the electrode was left at the injection site for 5 min after the virus injection was completed. The coordinates were defined as dorso-ventral (DV) from the brain surface, medio-lateral (ML) from the midline and anterior-posterior (AP) from bregma (in mm).

For *in vivo* 2P calcium imaging and fiber photometry, rAAV-CaMKIIa-GCaMP6f-WPRE-pA (AAV-CaMKII-GCaMP6f, AAV2/9, 2.53 × 10^12^ g/ml, 200 nl) virus was delivered into the right ACC (AP, +0.38 mm; ML, −0.25 mm; DV, −1.12 mm) of C57BL/6J mice. For chemogenetic manipulation, Cre-dependent virus rAAV-Ef1α-DIO-hM4D(Gi)-mCherry-WPRE-pA (AAV-DIO-hM4Di-mCherry, AAV2/9, 3.69 × 10^13^ vg/ml, 150 nl) or the virus rAAV-Ef1α-DIO-hM3D(Gq)-mCherry-WPRE-pA (AAV-DIO-hM3Dq-cherry, AAV2/9, 3.69 × 10^13^ vg/ml, 150 nl) was delivered into the right ACC of *CaMKII-Cre* mice, and CNO (5 mg/kg, MCE) was injected intraperitoneally 30 min before behavioral testing 3 weeks after virus injection. The CNO concentration was selected based on previous work using DREADD-based chemogenetics in mice that reported 3–5 mg/kg as an effective concentration for intraperitoneal CNO application (Jendryka et al., 2019). Many other studies using chemogenetic experiments in mice also reported a concentration of 5 mg/kg CNO (Milosavljevic et al., 2016; Zink et al., 2018; Traut et al., 2023). The rAAV-Ef1α-DIO-mCherry-WPRE-pA (AAV-DIO-mCherry, AAV2/8, 8.93 × 1,012 vg/ml) virus was used as a control (AP, +0.38 mm; ML, −0.25 mm; DV, −1.12 mm). To knockdown the expression of LCN2, the virus AAV-U6-shRNA (LCN2)-CMV-EGFP (1.0 × 10^12^ vg/ml, 250 nl) virus was introduced into the right ACC of SNI 2W mice (AP, +0.38 mm; ML, −0.25 mm; DV, −1.12 mm). AAV-U6-shRNA(scramble)-CMV-EGFP (2.0 × 10^12^ vg/ml, 250 nl) virus was used as a control (AP, +0.38 mm; ML, −0.25 mm; DV, −1.32 mm). All mice were transcranial perfused with ice-cold 0.9% saline followed by 4% PFA. Images of the signal expression were acquired with a confocal microscope LSM880 or LSM980 microscope. Animals with missed injections were excluded.

### Fiber photometry

Following AAV-CaMKIIa-GCaMP6f virus injection, an optical fiber [200 mm O.D., 0.37 numerical aperture (NA); Inper, Hangzhou, China] was placed in a ceramic ferrule and inserted toward the right ACC through the craniotomy. The ceramic ferrule was supported with three skull-penetrating M1 screws and dental acrylic. After the virus was expressed for 3 weeks, the optical-fiber-based Ca^2+^ signals of the ACC^Glu^ neuron population were detected by a custom-built setup (Thinkertech, Nanjing, China) during a pain threshold test. To excite GCaMP6f fluorescence, a 488-nm LED light beam (30 μW, Cree XPE LED, Coherent as a driver) was reflected by a dichroic mirror (MD498, Thorlabs) and coupled to an optical commutator (Doris Lenses) after focusing through a 20× objective lens (0.4 NA, Olympus). The light intensity at the tip of the fiber was 0.03 mW. Bandpass filtered (MF525-39, Thorlabs) light was collected by a photomultiplier tube (H10721-210, Hamamatsu) and then converted from the photomultiplier tube current output to voltage signals by an amplifier (C7319, Hamamatsu). A real-time processor including a Power 1401 digitizer and Spike2 software (CED, Cambridge, UK) was used to record the converted signal as a digitized signal. Ca^2+^ signals were sampled at 100 Hz through customized acquisition software written in LabView (National Instrument, USA). Behavioral videos were recorded with a video camera. Behavioral videos and neuronal Ca^2+^ signals were recorded simultaneously. Calcium signal analysis was conducted using Matlab toolkit OpSignal. For the chart or heatmaps of changes in Ca^2+^ signals, the ΔF/F (%) values were calculated as (F_signal_ − F_baseline_) / F_baseline_ × 100, where F_baseline_ is the mean of GCaMP6f signal for 2 s before time zero (von Frey stimulus initiation) and F_signal_ is the GCaMP6f signal for the entire session. A custom MATLAB script developed by ThinkerTech was used to form typical traces.

### *In vivo* 2P calcium imaging

Mice were first rapidly anesthetized with isoflurane, then deeply anesthetized with sodium pentobarbital (20 mg/kg, intraperitoneal injection), and immobilized on the stereotaxic instrument. Next, a solution of 2% iodophor and 75% alcohol was used as a disinfectant in the targeted brain region. The scalp and periosteum covering the dorsal skull were removed. A 3 × 3 mm piece of skull was removed with a dental drill and positioned on the right ACC according to the stereotactic coordinates (AP, +0.38 mm; ML, −0.25 mm; DV, −1.12 mm). It was determined that the dura was located over the ACC; during this procedure, the dura was left intact and moistened with saline. Trace bleeding was absorbed by Murad (Fukangsen). Following craniectomy, the right ACC was injected with 200 nl of AAV-CaMKIIa-GCaMP6f for 2P calcium imaging investigations. After that, a smaller circular coverslip (3 mm, Bellco Glass Inc.) was introduced to provide room for the craniotomy, and the region was covered with 1.2% agarose. Finally, dental adhesive and 3M tissue adhesive were used to attach a specially made aluminum head plate to the skull (Japan).

Mice have been accustomed to the imaging environment prior to imaging to reduce anxiety-related behaviors associated with the head fixation procedure. Mice recovered after cranial window surgery (2–4 weeks). *In vivo* 2P imaging was performed primarily on a dedicated 2P microscope (FVMPE-RS, Olympus, Japan) coupled with a Macrotek Deep Vision laser (Spectra-Physics) and a scanning galvanometer. The GFP, GCaMP6f, or YFP lasers had common power at 920 nm for patterning (10% of the laser transmission; PMT voltage 380 V), a common intensity on a dichroic pattern at 575 nm, and a subsequent emission filter. Previously, GFP or YFP was captured at 525/70 nm (green channel). All imaging tests began with a series of low-magnification images that were scanned in the z-direction. These images were combined with counts of vascular markers and cranial windows to determine the same location online continuously throughout time. Mice from comparable trials using clear glass windows on imaging day were covered.

Imaging depth of 2P microscopy can commonly reach ∼500 μm for *in vivo* mouse brain imaging (with the surface of the cerebral cortex as the zero point), and it is widely used for cortical imaging (Cheng et al., 2020; Takasaki et al., 2020). In this study, we injected AAV-CaMKII-GCaMP6f virus into the ACC at a depth of −1.12 mm (using the skull surface as the zero point) in mice. Skull thickness is about 0.3 mm in mice, and the diffusion diameter of 200 nl virus is about 0.5 mm, so the neurons expressing the virus are located within the range of sensitivity for 2P imaging, and the video images we collected can clearly show virus expression.

### Brain slice preparation

Acute brain slices were prepared as previously described. Mice were deeply anesthetized by an intraperitoneal injection of pentobarbital sodium (2% w/v) and intracardially perfused with ice-cold oxygenated modified N-methyl-D-glucamine artificial cerebrospinal fluid (NMDG ACSF) that contained (in mM) 30 NaHCO_3_, 2.5 KCl, 93 NMDG, 1.2 NaH_2_PO_4_, 25 glucose, 2 thiourea, 20 HEPES, 3 Na-pyruvate, 10 MgSO_4_, 5 Na-ascorbate and 0.5 CaCl_2_, and 3 glutathione (pH: 7.3-7.4, osmolarity: 300–310 mOsm/kg). Coronal slices (300 μm) that contained the ACC were sectioned on a vibrating microtome (VT1200s, Leica, Germany) at a rate of 0.18 mm/s. The sectioned brain slices were initially incubated in NMDG ACSF for 12–15 min at 33°C, followed by N-2-hydroxyethylpiperazine-N-2-ethanesulfonic acid (HEPES) ACSF that contained (in mM) 1.2 NaH_2_PO_4_, 2.5 KCl, 92 NaCl, 5 Na-ascorbate, 2 CaCl_2_, 30 NaHCO_3_, 3 Na-pyruvate, 20 HEPES, 3 GSH, 2 MgSO_4_, 2 thiourea, and 25 glucose (pH: 7.3–7.4, osmolarity: 300–310 mOsm/kg) for at least 1 h at 28°C. For whole cell recording, we transferred the brain slices to a slice chamber (Warner Instruments, USA) continuously perfused with standard ACSF solution (28°C) that contained (in mM) 3 KCl, 10 glucose, 3 HEPES, 129 NaCl, 1.3 MgSO_4_, 2.4 CaCl_2_, 20 NaHCO_3_, and 1.2 KH_2_PO_4_ (pH: 7.3–7.4, osmolarity: 300–310 mOsm/kg) at a rate of 2.5-3 ml/min. An in-line solution heater (TC-344B, Warner Instruments, USA) was used to maintain the temperature of the standard ACSF.

### Whole-cell patch-clamp recordings

To visualize neurons in the ACC region, we used a water-immersion objective (40×) on an upright microscope (BX51WI, Olympus, Japan), which was equipped with interference contrast (IR/DIC) and an infrared camera connected to the video monitor. Whole-cell patch-clamp recordings were obtained from visually identified the right ACC neurons. A four-stage horizontal puller (P1000, Sutter Instruments, USA) was used to obtain patch pipettes that were pulled from borosilicate glass capillaries (outer diameter: 1.5 mm, VitalSense Scientific Instruments Co., Ltd., Wuhan, China). The signals were acquired after being digitized at 10 kHz and low-pass filtered at 2.8 kHz via a Multiclamp 700B amplifier. The data were collected from the neurons with the appropriate input resistance (more than 100 MΩ) and series resistance (less than 30 MΩ). Experimental recording was immediately terminated when the series resistance changed by more than 20% during recording. The current-evoked firing was recorded in current-clamp mode (I = 0 pA) using pipettes filled with potassium gluconate-based internal solution resistance containing (in mM): 130 K-gluconate, 10 HEPES, 2 MgCl_2_, 5 KCl, 0.6 EGTA, 2 Mg-ATP, and 0.3 Na-GTP (pH: 7.2, osmolality: 285–290 mOsm/kg). The threshold current of the action potential was defined as the minimum current to elicit an action potential.

### *In vivo* pharmacological methods

A cannula (internal diameter 0.25 mm, RWD) was initially implanted into the right ACC of an anesthetized mouse that had been immobilized in a stereotactic frame. The cannula was supported with three skull-penetrating M1 screws and dental acrylic. An internal stainless-steel injector attached to a 10 μl syringe (Hamilton) and an infusion pump (micro 4, WPI, USA) was inserted into the guide cannula and used to infuse LCN2 mAb (0.5 μg/300 nl/day, R&D system), Isotype mAb (0.5 μg/300 nl/day, R&D system), rmLCN2 (1 μg/300 nl/day, R&D system) or ACSF (300 nl/day) into the right ACC at a flow rate of 150 nl/min. Isotype mAb is widely used as an isotype control in control experiments of mice (Shashidharamurthy et al., 2013; Deczkowska et al., 2021; Grubišić et al., 2022). Mice were administrated with LCN2 mAb, Isotype mAb, or ACSF starting at the 8 days after SNI treatment until the 14 days. Similarly, rmLCN2 or ACSF was injected into the right ACC of naïve mice for five consecutive days. The injector was slowly withdrawn 2 min after the infusion and the pain testing was performed roughly 0.5 h after the infusion.

### Western blotting method

For SNI 2W mice and C57 mice, unilateral right-sided ACC tissue was obtained by taking 300 μm-thick sections on a vibrating microtome. Tissue was homogenized in ice-cold RIPA lysis buffer (BL504A, Biosharp, Hefei, China) containing the protease inhibitor benzoyl fluoride (PMSF) (BL507A, Biosharp, Hefei, China) using a tissue grinder (Jingxin, Shanghai, China) and then lysed on ice for 10 min. at 4°C by centrifugation at 12,000 *g* for 15 min. The extracted proteins were denatured by boiling in a constant temperature metal bath at 98°C (10 min). A total of 20 μg of the resulting proteins were subjected to SDS-PAGE gel electrophoresis and then transferred to polyvinylidene difluoride (Bio-Rad). After blocking with 5% skimmed milk, the membranes were incubated overnight at 4°C with primary antibodies, including anti-LCN2 (1:1,000, rabbit, Proteintech, 26991-1-AP), β-actin (1:1,000, Absin, mouse, abs137975). Peroxidase-labeled secondary antibodies (1:5,000, Thermo Scientific) were incubated for 90 min at room temperature. Protein bands were visualized by chemiluminescence and quantified using ImageJ software. LCN2 levels were normalized to β-actin levels.

### Statistical analysis and drugs

GraphPad Prism 9 (GraphPad Software, Inc., USA) were used for the statistical analyses and graphing. Offline analysis of the data obtained from electrophysiological recordings was conducted using Clampfit software version 10.6 (Axon Instruments, Inc., USA). Animals were randomly or pseudo-randomly assigned to experimental groups, which minimized the influence of other variables on the experimental outcome. The normality of data was checked using the Shapiro–Wilk test. We conducted statistical comparisons between two groups using unpaired Student’s *t*-tests. Two-way analysis of variance (ANOVA) and Bonferroni *post hoc* analyses were used in analyses with multiple experimental groups. Mann–Whitney *U* test was used for data with non-normal distributions. Data are shown as individual values or expressed as the mean ± SEM, and significance levels are indicated as *P < 0.05, ^**^P < 0.01, and ^***^P < 0.001, and not significant (n.s.). *P*-values are not provided as exact values when they less than 0.0001. All statistical tests, significance analyses, number of individual experiments (*n*) and other relevant information for data comparison are specified in Supplementary Table 1. Unless otherwise stated, all drugs were purchased from Sigma-Aldrich (USA). CNO was obtained from MedChemExpress (China).

## Results

### ACC^Glu^ neuronal activity is enhanced in mice with SNI-induced chronic pain

**FIGURE 1:**
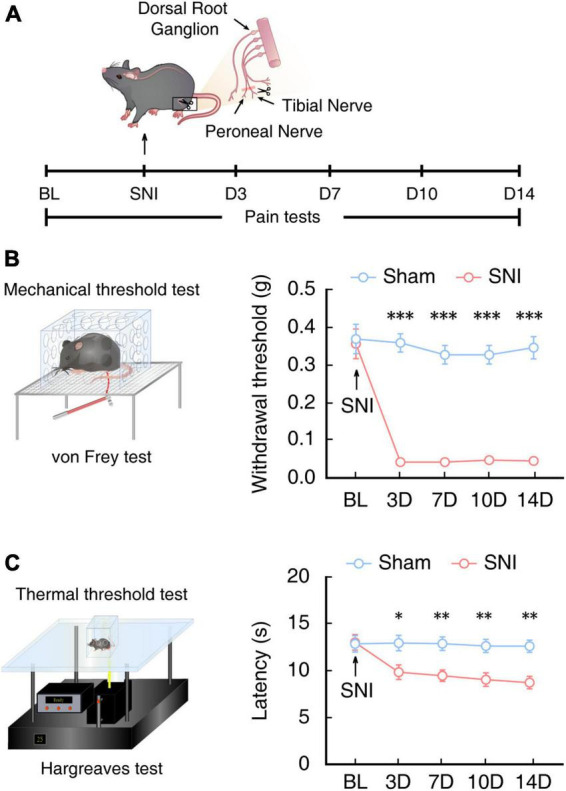
Assessment of pain sensitivity behaviors in mice at 2 weeks post SNI. **(A)** Schematic for inducing neuropathic pain and behavioral tests of mice. **(B)** Schematic for the von Frey test to assess the mechanical nociceptive threshold (left) and time course of mechanical nociceptive threshold of injured left hindpaws in SNI mice or sham mice (right, *n* = 8 mice per group, *F*_(1,70)_ = 249.3, *P* < 0.0001, two-way ANOVA with Bonferroni *post hoc* analysis). Arrows indicate SNI or sham surgery on 0 day. BL, baseline. **(C)** Schematic for the Hargreaves test to assess thermal pain thresholds (left) and the time course of thermal pain thresholds of injured left hindpaws in SNI mice or sham mice (right, *n* = 8 mice per group, *F*_(1,70)_ = 34.81, *P* < 0.0001, two-way ANOVA with Bonferroni *post hoc* analysis). Arrows indicate SNI or sham surgery on day 0 of surgery. BL, baseline. All data are presented as mean ± SEM. *P < 0.05; ^**^P < 0.01; ^***^P < 0.001.

To investigate the circuitry mechanisms underlying chronic pain, we first established a mouse model of chronic pain by SNI in male C57BL/6J mice (Bourquin et al., 2006), as previously described (Figure 1A). At 14 days after SNI (SNI 2W), mechanical nociceptive thresholds of the mice were significantly lower under von Frey stimuli compared with that in sham control mice (Figure 1B). We also found that SNI 2W mice had significantly lower thermal threshold in Hargreaves tests than that of sham mice (Figure 1C).

**FIGURE 2:**
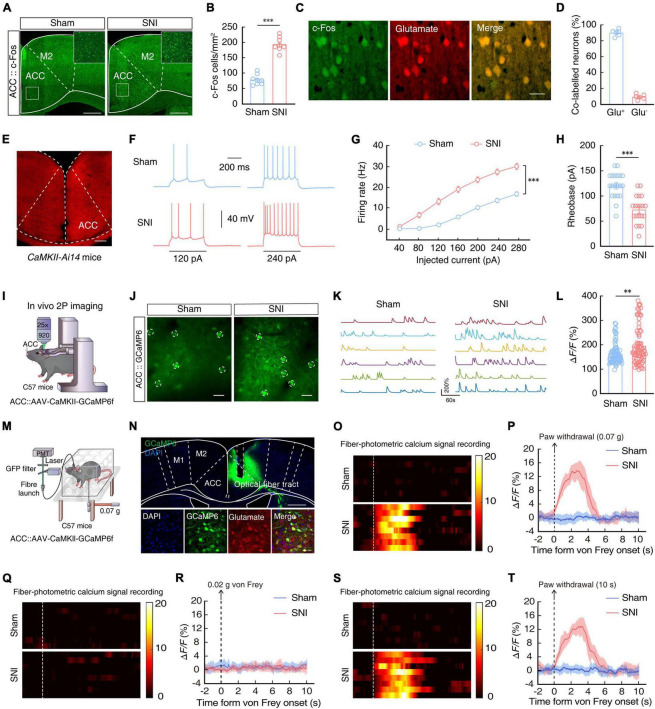
Enhanced ACC^Glu^ neuronal activity in SNI 2W mice. Typical images **(A)** and combined data (**B**, *n* = 8 slices per group; *t*_14_ = 11.47, *P* < 0.0001, two-tailed unpaired *t*-test) showing the expression of c-Fos in the contralateral ACC of SNI 2W or sham mice. Scale bars, 300 and 50 μm (enlargement). **(C)** Immunofluorescence staining images demonstrating the co-labeling of c-Fos-positive neurons (green) with glutamate immunofluorescence (red). Scale bar, 20 μm. **(D)** Summary data showing glutamate-positive neurons expressing c-Fos (left) and glutamate-negative neurons expressing c-Fos (right) are shown in the contralateral ACC of SNI 2W or sham mice (*n* = 6 mice per group). **(E)** Images of the sample of ACC neurons in a *CaMKII-Ai14* mouse. Scale bar, 200 μm. Representative traces **(F)** and summarized data (**G**, *n* = 20 cells per group, *F*_(1,38)_ = 58.98, *P* < 0.0001, two-way ANOVA with Bonferroni *post hoc* analysis) of action potentials recorded from tdTOM^+^ neurons in the ACC slices of SNI 2W and sham mice. **(H)** Statistical analysis of the rheobase of the spike recorded in contralateral ACC^Glu^ neurons (*n* = 20 cells per group, *t*_38_ = 5.781, two-tailed unpaired *t*-test). **(I)** Schematic diagram of *in vivo* two-photon (2P) calcium imaging in head-constrained mice. **(J)** Representative images of 2P CaMKII-GCaMP6f imaging fields from sham and SNI 2W mice. Scale bars, 20 μm. **(K)** Example spontaneous Δ*F/F* time-series traces from the imaging fields in panel **(J)**. **(L)** Average spontaneous calcium responses in GCaMP6^+^ contralateral ACC^Glu^ neurons (*n* = 80 cells per group, *U* = 2,424, *P* = 0.0079, Mann–Whitney *U* test). **(M)** Schematic illustration of the fiber optic device used to measure ACC^Glu^ neuronal calcium. **(N)** Representative images verifying GCaMP6f expression in glutamatergic neurons and light fiber bundles above the ACC (top). Images depict the overlap between cells expressing GCaMP6f (green) and glutamate-resistant positive cells (red) (bottom). Scale bars, 500 μm (top) and 20 μm (bottom). Heat map illustration **(O)** and mean Δ*F/F* **(P)** of GCaMP6f signals show a rapid increase in Ca^2+^ signals in ACC^Glu^ neurons after 0.07-g von Frey stimulation in SNI 2W mice compared to sham mice (*n* = 8 mice per group). Color scale on the right indicates Δ*F/F* (%). Heat map illustration **(Q)** and mean Δ*F/F* **(R)** of GCaMP6f signals show no increase in Ca^2+^ signals in ACC^Glu^ neurons after 0.02-g von Frey stimulation in SNI 2W mice compared to sham mice (*n* = 8 mice per group). Color scale on the right indicates Δ*F/F* (%). Heat map illustration **(S)** and mean Δ*F/F* **(T)** of GCaMP6f signals show a rapid increase in Ca^2+^ signals in ACC^Glu^ neurons at thermal stimulation threshold of 10 s for paw withdrawal in SNI 2W mice compared to sham mice (*n* = 8 mice per group). Color scale on the right indicates Δ*F/F* (%). All data are presented as mean ± SEM. ^**^P < 0.01; ^***^P < 0.001.

The ACC is well-established as a major brain region responsible for processing pain signals, integrating pain-related information from the spinal cord, thalamus, and other regions (Bliss et al., 2016; Meda et al., 2019; Zhu et al., 2021). Immunofluorescence staining for c-Fos, an immediate-early gene marker of neural activity, in mouse brain slices revealed that c-Fos^+^ neurons were significantly more abundant in the ACC of SNI 2W mice than in sham mice (Figures 2A, B). Moreover, approximately 90% of c-Fos^+^ neurons also co-localized with neurons labeled by glutamate antibody in SNI 2W mice (Figures 2C, D).

To characterize the activity in these glutamatergic neurons of the ACC (ACC^Glu^), we performed whole-cell patch-clamp electrophysiological recordings in ACC^Glu^ neurons expressing the tdTomato reporter in brain slices of *CaMKII-Ai14* mice (Figure 2E). We observed that the evoked spike number was increased (Figures 2F, G) while rheobase of the spike was decreased (Figure 2H) in SNI 2W mice compared with that in sham mice.

*In vivo* 2P calcium imaging of ACC^Glu^ neurons in sham and SNI 2W mice was next used to verify these results. To this end, we injected AAV-CaMKII-GCaMP6f (GCaMP6f) virus into the ACC (Figure 2I) and confirmed the CaMKII-driven expression of GCaMP6f at 3 weeks post injection (Figure 2J and Supplementary Video 1). Comparison with sham mice indicated that fluorescence signal from the Ca^2+^ reporter was significantly higher in ACC^Glu^ neurons of SNI 2W mice (Figures 2K, L).

To directly determine whether ACC^Glu^ neurons were sensitized to subthreshold stimuli, we infused the ACC of SNI 2W and sham mice with AAV-CaMKII-GCaMP6f and used optical fiber photometry to measure Ca^2+^ signals in ACC^Glu^ neurons under von Frey stimuli (Figures 2M, N). We found that the threshold was approximately 0.05 g, and therefore used the 0.07-g von Frey filament, which is close to 0.05 g, but more likely to induce withdrawal or licked paw responses in SNI 2W mice. However, 0.07 g was a sub-threshold stimulus for sham mice, and so we used this stimulus to detect changes in pain sensitivity in SNI 2W mice. We found that Ca^2+^ signals in ACC^Glu^ neurons rapidly increased following 0.07-g von Frey stimuli to injured left hindpaws of SNI 2W mice, compared with that recorded in sham mice (Figures 2O, P). We examined pain sensitivity in SNI 2W mice using a lower von Frey stimulus of 0.02 g. The results showed that 0.02 g von Frey stimulus failed to induce either withdrawal or licked paw responses in both sham and SNI 2W mice, and no transient enhancement of calcium signals was detected upon stimulation (Figures 2Q, R). In addition, we also tested the response of ACC^Glu^ neurons to thermal stimulation. The results showed that 10 s of thermal stimulation could induce a withdrawal or licked paw response, as well as a transient increase in calcium activity of ACC^Glu^ neurons, in SNI 2W mice but not in sham mice (Figures 2S, T). These results indicated that this threshold was below that of sham mice, but higher than that of SNI 2W mice, and that ACC^Glu^ neurons could also respond to thermal stimulation. These cumulative data suggested that ACC^Glu^ neuronal excitability was enhanced in mice with SNI-induced chronic pain.

### Chemogenetic inhibition of ACC^Glu^ neuronal activity alleviates chronic pain

**FIGURE 3:**
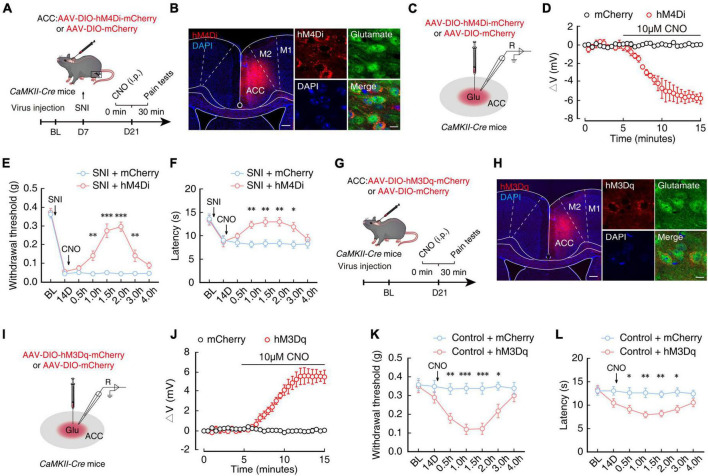
Chemogenetic inhibition of ACC^Glu^ neuronal activity alleviates pain sensitization. **(A)** Schematic diagram of experimental procedures for manipulation of chemogenetic inhibition and behavioral testing in SNI 2W mice. **(B)** Representative image showing the injection site of AAV-DIO-hM4Di-mCherry (hM4Di) or AAV-DIO-mCherry (mCherry) in the ACC of *CaMKII-Cre* mice (left). Image (right) shows that hM4Di-labeled neurons within the ACC are primarily co-localized with glutamate immunofluorescent signals (green). Scale bars, 200 μm (left) and 10 μm (right). **(C)** Diagram of virus injection and logging configuration. **(D)** Whole-cell recordings of acute sections showing the effect of CNO on hM4Di- or mCherry-expressing ACC^Glu^ neurons (*n* = 5 mice per group). Effect of chemical genetic inhibition of ACC^Glu^ neurons on mechanical nociceptive thresholds (**E**, *n* = 8 mice per group, *F*_(1,112)_ = 116.3, *P* < 0.0001, two-way ANOVA with Bonferroni *post hoc* analysis) and thermal pain thresholds (**F**, *n* = 8 mice per group, *F*_(1,112)_ = 28.56, *P* < 0.0001, two-way ANOVA with Bonferroni *post hoc* analysis) of SNI 2W mice after mCherry or hM4Di infusion in the right ACC. **(G)** Schematic diagram of experimental procedures for manipulation of chemogenetic activation and behavioral testing in naïve mice. **(H)** Representative image showing the injection site of AAV-DIO-hM3Dq-mCherry (hM3Dq) or mCherry in the ACC of *CaMKII-Cre* mice (left). Image (right) shows that hM3Dq-labeled neurons within the ACC are primarily co-localized with glutamate immunofluorescent signals (green). Scale bars, 200 μm (left) and 10 μm (right). **(I)** Diagram of virus injection and logging configuration. **(J)** Whole-cell recordings of acute sections showing the effect of CNO on hM3Dq- or mCherry-expressing ACC^Glu^ neurons (*n* = 8 mice per group). Effect of chemogenetic activation of ACC^Glu^ neurons on mechanical nociceptive thresholds (**K**, *n* = 8 mice per group, *F*_(1,98)_ = 53.69, *P* < 0.0001, two-way ANOVA with Bonferroni *post hoc* analysis) and thermal pain thresholds (**L**, *n* = 8 mice per group, *F*_(1,98)_ = 39.66, *P* < 0.0001, two-way ANOVA with Bonferroni *post hoc* analysis) of naïve mice after mCherry or hM3Dq infusion in the ACC. All data are presented as mean ± SEM. *P < 0.05; ^**^P < 0.01; ^***^P < 0.001.

To further explore how ACC^Glu^ neurons participate in pain sensitization, we probed the relationship between neuronal activity and chronic pain by selectively inhibiting ACC^Glu^ neurons in *CaMKII-Cre* mice through ACC injection with AAV-DIO-hM4Di-mCherry to induce Cre-dependent expression of hM4Di (Figures 3A, B) and intraperitoneal injection of its ligand, clozapine-N-oxide (CNO, 5 mg/kg). Electrophysiological recordings in brain slices revealed that the resting membrane potential (RMP) in hM4Di-mCherry^+^ ACC^Glu^ neurons was significantly hyperpolarized after 10 μM CNO perfusion compared to that in SNI 2W mice expressing only the mCherry reporter (Figures 3C, D). In addition, we found that selective inhibition of ACC^Glu^ neuronal activity by CNO injection could also significantly restore the mechanical nociceptive and thermal pain thresholds in SNI 2W mice (Figures 3E, F).

In addition, we infused the ACC of naïve *CaMKII-Cre* mice with virus expressing chemogenetic excitatory hM3Dq (AAV-DIO-hM3Dq-mCherry) to selectively activate ACC^Glu^ neurons (Figures 3G, H). Using the same method as above, we found that RMP of ACC^Glu^ neurons expressing hM3Dq was depolarized after perfusion with 10 μM CNO (Figures 3I, J). Mechanical nociceptive threshold tests and thermal pain tests further indicated that pain sensitivity was increased in naïve mice with selectively activated ACC^Glu^ neurons by 5 mg/kg CNO injection (Figures 3K, L). These collective results suggested that enhanced ACC^Glu^ neuronal activity was required for the development of chronic pain.

### LCN2 expression is increased in the ACC after SNI 2W

**FIGURE 4:**
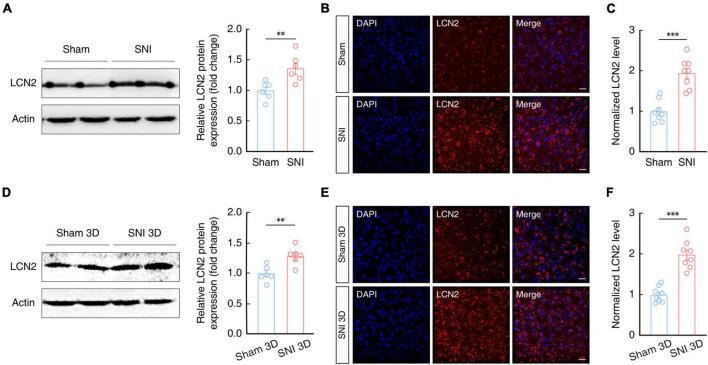
Increased LCN2 expression in the ACC of SNI 2W and SNI 3D mice. **(A)** Western blot analysis of LCN2 expression in the contralateral ACC of SNI 2W and sham 2W mice (*n* = 6 mice per group, *t*_10_ = 3.291, *P* = 0.0081, two-tailed unpaired *t*-test). Representative images **(B)** and quantitative analyses (**C**, *n* = 8 slices per group, *t*_14_ = 5.872, *P* < 0.0001, two-tailed unpaired *t*-test) of immunostaining for LCN2 in the ACC of SNI 2W and sham 2W mice. Scale bars, 20 μm. **(D)** Western blot analysis of LCN2 expression in the contralateral ACC of SNI 3D and sham 3D mice (*n* = 6 mice per group, *t*_10_ = 3.588, *P* = 0.0049, two-tailed unpaired *t*-test). Representative images **(E)** and quantitative analyses (**F**, *n* = 8 slices per group, *t*_14_ = 6.993, *P* < 0.0001, two-tailed unpaired *t*-test) of immunostaining for LCN2 in the ACC of SNI 3D and sham 3D mice. Scale bars, 20 μm. All data are presented as mean ± SEM. ^**^P < 0.01; ^***^P < 0.001.

Lipocalin-2 and its receptor, 24p3R, are known to play an active role in regulation of neuronal plasticity and behavioral responses (Ferreira et al., 2013; Jha et al., 2015; Dekens et al., 2021; Xiang et al., 2022). To explore whether the LCN2 contribute to the generation of chronic pain, we first examined LCN2 expression in the ACC of SNI 2W and sham mice by Western blotting. The results showed that LCN2 was indeed expressed at significantly higher levels in the ACC of SNI 2W mice than in sham mice (Figure 4A). Additionally, immunofluorescence staining further confirmed these results (Figures 4B, C). To investigate whether LCN2 expression is involved in both the development (i.e., early stage, 3D post SNI) and maintenance (late stage, 2W post SNI) of chronic pain. We quantified LCN2 expression levels in the ACC in SNI 3D mice. Western blotting showed that LCN2 levels were significantly higher in the ACC of SNI 3D mice compared to Sham 3D mice (Figure 4D), which was consistent with results of immunofluorescence staining image analysis (Figures 4E, F). These results further supported our conclusion that LCN2 participates in both the development and maintenance of chronic pain.

**FIGURE 5:**
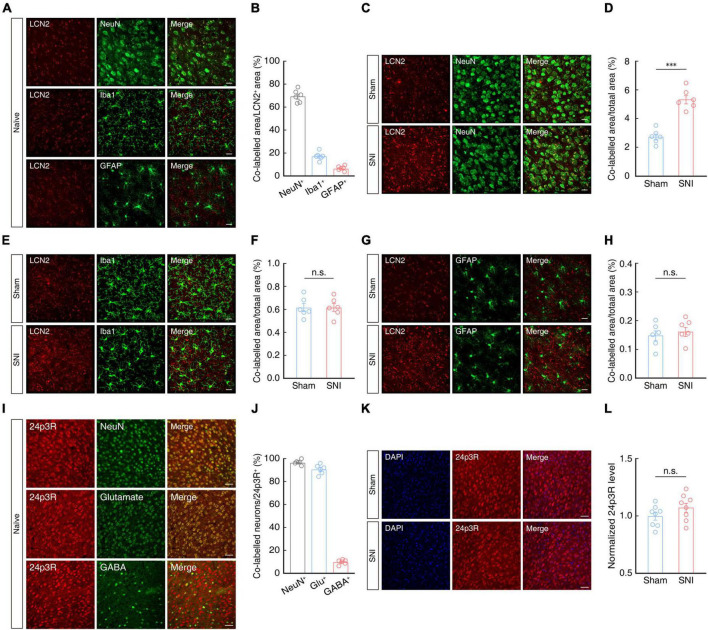
Cellular distribution of LCN2 in the ACC. Representative images **(A)** and statistical analysis **(B)** of LCN2 (red) co-localization with NeuN, Iba-1, or GFAP (green) in the ACC of naïve mice (*n* = 6 mice per group). Scale bars, 20 μm. Representative images **(C)** and summary data (**D**, *n* = 6 mice per group, *t*_10_ = 7.414, *P* < 0.0001, two-tailed unpaired *t*-test) of areas co-labeled with LCN2 (red) and NeuN (green) indicating LCN2 localization with neurons in the contralateral ACC of SNI 2W or sham mice. Scale bar, 20 μm. Representative images **(E)** and summary data (**F**, *n* = 6 mice per group, *t*_10_ = 0.0171, *P* = 0.9867, two-tailed unpaired *t*-test) of areas co-labeled with LCN2 (red) with Iba-1 (green), indicating LCN2 co-localization with microglia in the contralateral ACC of SNI 2W or sham mice. Scale bar, 20 μm. Representative images **(G)** and summary data (**H**, *n* = 6 mice per group, *t*_10_ = 0.5748, *P* = 0.5781, two-tailed unpaired *t*-test) of areas co-labeled with LCN2 (red) and GFAP (green), indicating LCN2 co-localization with astrocytes in the contralateral ACC of SNI 2W or sham mice. Scale bar, 20 μm. Representative images **(I)** and statistics data **(J)** show co-localization of 24p3R (red) with NeuN, glutamate, and GABA in the ACC (*n* = 6 slices per group). Scale bars, 50 μm. Representative images **(K)** and quantitative analyses (**L**, *n* = 8 slices per group, *t*_14_ = 1.449, *P* = 0.1694, two-tailed unpaired *t*-test) of immunostaining for 24p3R in the ACC of SNI 2W and sham mice. Scale bars, 50 μm. All data are presented as mean ± SEM. ^***^P < 0.001; n.s., not significant.

Previous studies have reported that LCN2 protein is secreted by neurons (Jha et al., 2015; Nagase and Tohda, 2021) and glial cells (Bi et al., 2013; Jang et al., 2013; Staurenghi et al., 2021; Weng et al., 2021). To determine the cellular distribution of LCN2 protein in the ACC of mice, we detected LCN2 by immunofluorescence staining and found that it mainly co-localized with neurons, and to a small extent with microglia and astrocytes in the ACC of naïve mice (Figures 5A, B). In the ACC of SNI 2W mice, we observed a significant increase in areas co-labeled with antibodies for LCN2 and NeuN neuronal marker compared to that in sham 2W mice (Figures 5C, D), while no significant change in LCN2 expression levels were detected in microglia or astrocytes (Figures 5E–H).

We then examined the source and expression levels of the LCN2 receptor, 24p3R, by immunofluorescence staining of the ACC in mice, which revealed that signal for 24p3R primarily co-localized with NeuN signal, and that ∼90% of these 24p3R^+^ neurons also co-localized with signal for glutamate antibody in naïve mice (Figures 5I, J). Notably, 24p3R protein levels in ACC were not significantly different between SNI 2W and sham mice (Figures 5K, L).

**FIGURE 6:**
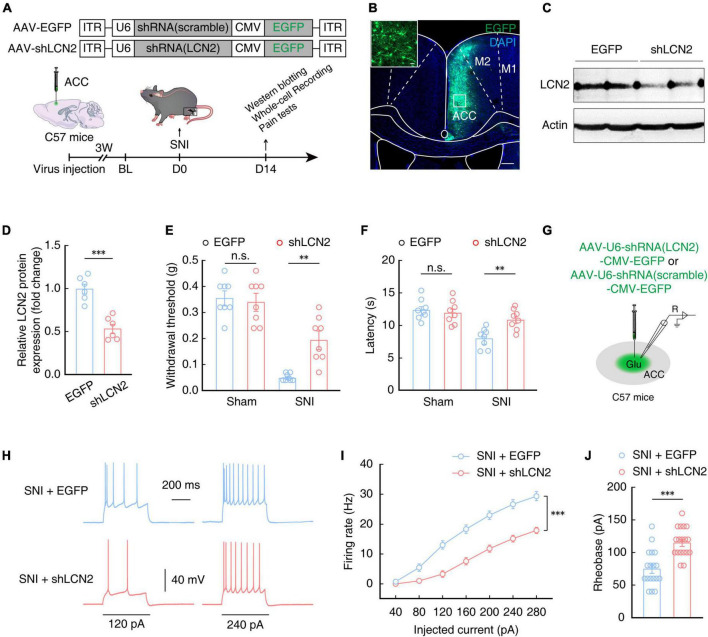
Lipocalin-2 knockdown reduces pain sensitivity and decreases ACC^Glu^ neuronal activity. **(A)** Schematic for a scramble (AAV-EGFP) or shLCN2 (AAV-shLCN2) virus injection and behavioral tests. **(B)** Typical images showing the expression of virus in the right ACC of SNI 2W mice. Scale bars, 200 and 20 μm (enlargement). **(C,D)** Western blotting of LCN2 expression in the right ACC of SNI 2W mice lysates after AAV-EGFP (EGFP) or AAV-shLCN2 (shLCN2) infusion (*n* = 6 mice per group, *t*_10_ = 5.805, *P* = 0.0002, two-tailed unpaired *t*-test). Effect of LCN2 knockdown on mechanical nociceptive thresholds (**E**, *n* = 8 mice per group, *F*_(1,28)_ = 6.962, *P* = 0.0134, two-way ANOVA with Bonferroni *post hoc* analysis) and thermal pain thresholds (**F**, *n* = 8 mice per group, *F*_(1,28)_ = 4.569, *P* = 0.0414, two-way ANOVA with Bonferroni *post hoc* analysis) in SNI 2W or sham mice after EGFP or shLCN2 infusion in the ACC. **(G)** Diagram of virus injection and logging configuration. Representative traces **(H)** and summarized data (**I**, *n* = 18 cells per group, *F*_(1,34)_ = 34.58, *P* < 0.0001, two-way ANOVA with Bonferroni *post hoc* analysis) of action potentials recorded from ACC^Glu^ neurons of SNI 2W mice after EGFP (SNI + EGFP) or shLCN2 (SNI + shLCN2) infusion. **(J)** Statistical analysis of the rheobase of the spike recorded in contralateral ACC^Glu^ of SNI 2W mice after EGFP (SNI + EGFP) or shLCN2 (SNI + shLCN2) infusion (*n* = 18 cells per group, *t*_34_ = 4.751, *P* < 0.0001, two-tailed unpaired *t*-test). All data are presented as mean ± SEM. ^**^P < 0.01; ^***^P < 0.001; n.s., not significant.

Lipocalin-2 protein is secreted from neurons (Jha et al., 2015; Nagase and Tohda, 2021) as well as glial cells (Bi et al., 2013; Jang et al., 2013; Staurenghi et al., 2021; Weng et al., 2021). In order to explore whether up-regulation of LCN2 in the ACC is involved in the generation of chronic pain, we next constructed generated a broad-spectrum virus expressing short hairpin RNAs (shRNAs) to knock down LCN2 expression. The SNI model was established at 3 weeks after ACC injection with AAV-U6-shRNA (LCN2)-CMV-EGFP (shLCN2) or AAV-U6-shRNA(scramble)-CMV-EGFP (EGFP) (Figures 6A, B). Western blotting analysis confirmed that LCN2 protein levels were significantly decreased in SNI 2W with LCN2 knockdown mice compared to that in mice infected with the AAV-EGFP control (Figures 6C, D). The results of mechanical nociceptive and thermal pain threshold tests showed that SNI 2W mice with ACC injection of AAV-shLCN2 had significantly higher thresholds and longer latency than SNI 2W mice injected with the AAV-EGFP control virus (Figures 6E, F), supporting that LCN2 knockdown in the ACC could relieve pain perception in SNI 2W mice.

Electrophysiological recordings of ACC^Glu^ neurons in brain slices of SNI 2W mice injected with shLCN2 or EGFP control virus (Figure 6G) showed a decrease in the evoked spike number (Figures 6H, I) and an increase in rheobase of the spike (Figure 6J) in SNI 2W mice with LCN2 knockdown in the ACC compared with that in AAV-EGFP control mice. These above results showed that downregulation of LCN2 in the ACC could reduce the activity of ACC^Glu^ neurons, consequently relieving pain sensitivity in SNI 2W mice.

### Regulation of LCN2 levels in the ACC affects pain sensitivity

**FIGURE 7:**
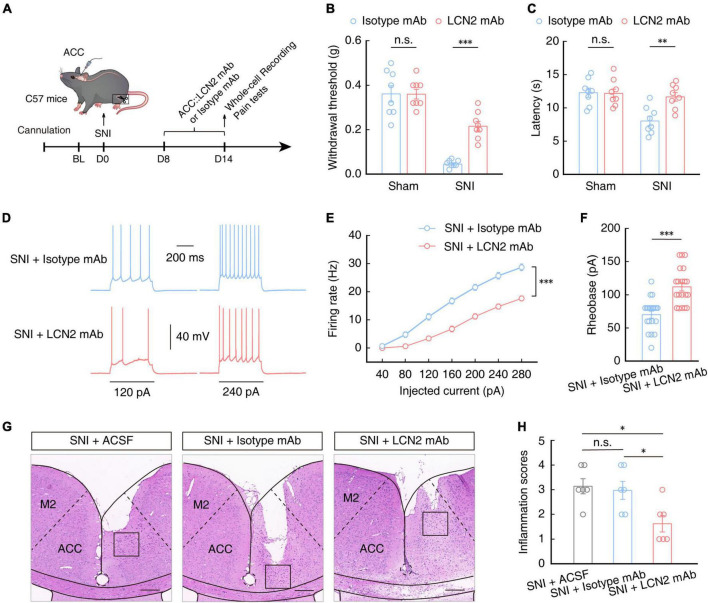
Pain sensitivity in SNI 2W mice treated with LCN2 mAb. **(A)** Schematic of ACC injected with neutralizing antibody targeting LCN2 (LCN2 mAb) or Isotype mAb in SNI 2W or sham mice. Mechanical nociceptive thresholds (**B**, *F*_(1,28)_ = 12.45, *P* = 0.0015, two-way ANOVA with Bonferroni *post hoc* analysis) and thermal pain thresholds (**C**, *F*_(1,28)_ = 6.400, *P* = 0.0173, two-way ANOVA with Bonferroni *post hoc* analysis) in SNI 2W or sham mice with ACC infusion of LCN2 or Isotype mAbs (*n* = 8 mice per group). Representative traces **(D)** and summarized data (**E**, *n* = 20 cells per group, *F*_(1,38)_ = 50.23, *P* < 0.0001, two-way ANOVA with Bonferroni *post hoc* analysis) of action potentials recorded in ACC^Glu^ neurons of SNI 2W mice brain slices incubated with LCN2 or Isotype mAbs. **(F)** Statistical analysis of the rheobase of the spike recorded in ACC^Glu^ of SNI 2W mice brain slices incubated with LCN2 or Isotype mAbs (*n* = 20 cells per group, *U* = 50, *P* < 0.0001, Mann–Whitney *U* test). Representative images **(G)** and quantitative analyses (**H**, *n* = 6 mice per group, *F*_(2,15)_ = 5, *P* = 0.0123, one-way ANOVA with Bonferroni *post hoc* analysis) of H&E staining of brain slices from the ACC of SNI 2W mice cannulated and continuously infused with ACSF, Isotype mAb, or LCN2 mAb. Scale bars, 200 μm. All data are presented as mean ± SEM. *P < 0.05; ^**^P < 0.01; ^***^P < 0.001; n.s., not significant.

To further characterize the role of LCN2 in the generation of chronic pain in SNI model mice, we implanted a catheter to administer a neutralizing antibody targeting LCN2 (LCN2 mAb) in the ACC of SNI 2W mice (Figure 7A). After 7 days of continuous LCN2 mAb administration, the mechanical nociceptive and thermal pain thresholds were significantly recovered in SNI 2W mice compared to those in SNI 2W mice treated with Isotype mAb (Figures 7B, C). Electrophysiological recordings in brain slices showed that the firing rate of evoked action potentials decreased (Figures 7D, E) while the rheobase increased (Figure 7F) in ACC^Glu^ neurons of SNI 2W mice treated with LCN2 mAb compared to the Isotype mAb control group, aligning well with our above results of LCN2 knockdown. We also detected the immune response to mAbs in SNI mice through H&E staining, which showed no significant difference in inflammatory cell infiltration between SNI mice treated with Isotype mAb and ACSF-treated controls (Figures 7G, H). Notably, inflammatory cell infiltration was also significantly lower in LCN2 mAb-treated SNI 2W mice than in Isotype mAb-treated controls (Figures 7G, H). These results suggested the absence of immune response to Isotype mAb.

**FIGURE 8:**
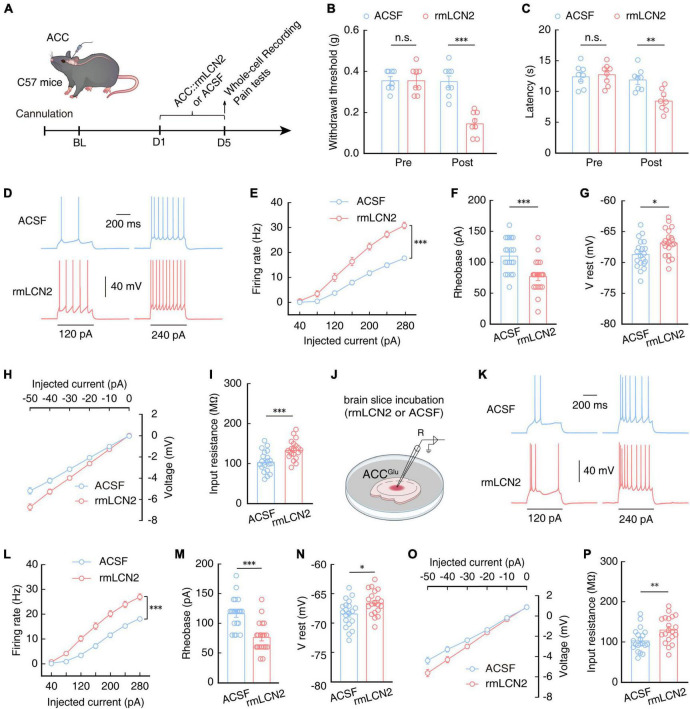
Increasing LCN2 levels in the ACC can enhance ACC^Glu^ neuronal excitability and induce pain sensitivity. **(A)** Schematic of right ACC injected with purified recombinant protein of LCN2 (rmLCN2) or ACSF in naïve mice. Effect of rmLCN2 on mechanical nociceptive thresholds (**B**, *n* = 8 mice per group, *F*_(1,28)_ = 21.48, *P* < 0.0001, two-way ANOVA with Bonferroni *post hoc* analysis) and thermal pain thresholds (**C**, *n* = 8 mice per group, *F*_(1,28)_ = 6.140, *P* = 0.0195, two-way ANOVA with Bonferroni *post hoc* analysis) in naïve mice with ACC infusion of rmLCN2 or ASCF. Representative traces **(D)** and summarized data (**E**, *n* = 20 cells per group, *F*_(1,38)_ = 36.60, *P* < 0.0001, two-way ANOVA with Bonferroni *post hoc* analysis) of action potentials recorded in ACC^Glu^ neurons following ACC infusion with rmLCN2 or ACSF in naïve mice. **(F)** Statistical analysis of the rheobase of the spike recorded in ACC^Glu^ neurons following ACC infusion with rmLCN2 or ACSF in naïve mice (*n* = 20 cells per group, *t*_38_ = 3.879, *P* < 0.0001, two-tailed unpaired *t*-test). **(G)** RMP recorded in ACC^Glu^ neurons following ACC infusion with rmLCN2 or ACSF in naïve mice (*n* = 20 cells per group, *t*_38_ = 2.671, *P* = 0.0111, two-tailed unpaired *t*-test). **(H)** Voltage-current plots of voltage responses to a stepwise series of hyperpolarizing currents (0 to –50 pA, –10 pA/step; duration: 500 ms) recorded in ACC^Glu^ neurons following ACC administration with rmLCN2 or ACSF in naïve mice (*n* = 20 cells per group). **(I)** Input resistance recorded in ACC^Glu^ neurons of naïve mice with ACC infusion of rmLCN2 or ACSF (*n* = 20 cells per group, *t*_38_ = 3.737, *P* = 0.0006, two-tailed unpaired *t*-test). **(J)** Schematic for incubating brain slices of naïve mice with rmLCN2 or ACSF. These samples were used for panels **(K–P)**. Representative traces **(K)** and summarized data (**L**, *n* = 20 cells per group, *F*_(1,38)_ = 31.89, *P* < 0.0001, two-way ANOVA with Bonferroni *post hoc* analysis) of action potentials recorded in ACC^Glu^ neurons in brain slices from panel **(J)**. **(M)** Statistical analysis of rheobase of spikes recorded in ACC^Glu^ neurons in brain slices from panel **(J)** (*n* = 20 cells per group, *t*_38_ = 4.832, *P* < 0.0001, two-tailed unpaired *t*-test). **(N)** RMP recorded in ACC^Glu^ neurons in brain slices from panel **(J)** (*n* = 20 cells per group, *t*_38_ = 2.672, *P* = 0.0110, two-tailed unpaired *t*-test). **(O)** Voltage-current plots of voltage responses to a stepwise series of hyperpolarizing currents (0 to –50 pA, –10 pA/step; duration: 500 ms) recorded in ACC^Glu^ neurons in brain slices from panel **(J)** (*n* = 20 cells per group). **(P)** Input resistance recorded in ACC^Glu^ neurons in brain slices from panel **(J)** (*n* = 20 cells per group, *t*_38_ = 2.777, *P* = 0.0085, two-tailed unpaired *t*-test). All data are presented as mean ± SEM. *P < 0.05; ^**^P < 0.01; ^***^P < 0.001; n.s., not significant.

To investigate whether exogenous LCN2 application affected pain sensitivity, we injected purified recombinant LCN2 (rmLCN2) protein into the ACC through an embedded catheter in naïve mice (Figure 8A). After 5 days of continuous rmLCN2 administration, both the mechanical nociceptive and thermal pain thresholds were significantly lower than those recorded in naïve mice following ACSF injection (Figures 8B, C). Subsequent electrophysiological recordings revealed an increase in the number (Figures 8D, E) and a decrease in rheobase of the evoked spike (Figure 8F), accompanied by depolarized RMP (Figure 8G) and increased membrane input resistance (Figures 8H, I) in ACC^Glu^ neurons of naïve mice with ACC administration of rmLCN2 compared to that in the ACSF control group. These results suggested that the membrane properties of ACC^Glu^ neurons change in response to repetitive administration of rmLCN2.

In order to characterize an acute response of rmLCN2 protein to ACC^Glu^ neurons, we incubated isolated brain slices with rmLCN2 for 30 min (Figure 8J), based on previous reports that indicated this strategy can accurately reflect the effects and stability of drugs on neuronal activity (Taura et al., 2004; Kim et al., 2005; Yao et al., 2010). Similar to the changes observed in ACC^Glu^ neurons following repeated administration of rmLCN2, we found that the membrane properties of ACC^Glu^ neurons exhibited similar changes in brain slices incubated with rmLCN2 (Figures 8K–P). These results suggest that rmLCN2 can induce an acute response in ACC^Glu^ neurons.

These cumulative results indicated that increasing LCN2 levels in the ACC can enhance ACC^Glu^ neuronal excitability and induce pain sensitivity, decreasing levels of LCN2 in the ACC can reduce glutamatergic neuron excitability and relieve pain in SNI 2W mice.

## Discussion

Chronic pain represents a major health care problem and seriously impacts quality of life for millions of patients. However, clinical analgesic drugs currently available for treating chronic pain remain unsatisfactory. To resolve this problem, numerous studies have explored the primary underlying mechanisms responsible for chronic pain. Previous studies have demonstrated that activation of the ACC through long-term potentiation contributes to chronic pain states (Li et al., 2010, 2021; Zhuo, 2014). In addition, peripheral immune cells and glial cells also participate in processing pathological pain (Sorge et al., 2015; Inoue and Tsuda, 2018; Tozaki-Saitoh and Tsuda, 2019; Finnerup et al., 2021). Although LCN2 is known to play a role in the development of inflammatory pain via activation of spinal microglia (Jha et al., 2014, 2015), it remains unclear whether LCN2 is also involved in regulating neural plasticity, a process fundamental to central sensitization during pain development. Our findings indicate that up-regulation of LCN2 in the ACC could indeed regulate neural plasticity in ACC^Glu^ neurons, ultimately driving the development and maintenance of chronic pain.

Lipocalin-2 secretion has been observed in a wide range of cell types such as reactive astrocytes (Bi et al., 2013; Staurenghi et al., 2021; Weng et al., 2021), endothelial vascular cells (Marques et al., 2008; Wang et al., 2015), activated microglia (Lee et al., 2007; Ip et al., 2011), and neurons (Jha et al., 2015; Nagase and Tohda, 2021; Xiang et al., 2022). We found that LCN2 is mainly distributed in neurons of the ACC, with strikingly lower immunofluorescent signal co-localizing with markers for microglia and astrocytes in the ACC of naïve mice. Furthermore, we observed that the area of LCN2/neuron co-localizations was significantly increased in the ACC of SNI 2W mice compared to that in sham 2W mice, while no significant change in LCN2 levels was observed in microglia or astrocytes. Since LCN2 upregulation was exclusively detected in ACC neurons at 2W post SNI surgery, and not in microglia or astrocytes, it is reasonable to conclude that LCN2 in ACC neurons participates in pain perception.

The involvement of inflammatory factors in the development of pain is well-established (Kavelaars and Heijnen, 2021; Niehaus et al., 2021). Various changes in the transcriptional and secretory profiles of immune cells are associated with chronic pain, including the release of tumor necrosis factor, various interleukins, schistocytes, ATP, and chemokines (Ren and Dubner, 2010; Zhang et al., 2017; Tozaki-Saitoh and Tsuda, 2019; Finnerup et al., 2021). However, the upstream factors that drive this intensified secretion of inflammatory factors remain largely unknown. The release of inflammatory factors by microglia and astrocytes can reportedly regulate neuronal plasticity in the ACC, promote the sustained excitation of neurons, and subsequently lead to of pain sensitization (Tsuda et al., 2017; Li et al., 2019). Therefore, it is plausible that the up-regulation of LCN2 may drive the release of such inflammatory factors to induce the development of chronic pain. Conversely, previous studies have shown that the up-regulation of inflammatory factors could also increase the expression of LCN2 (Bu et al., 2006; Cowland et al., 2006). It appears likely that the regulation of LCN2 and inflammatory factor recycling in the ACC may drive the chronicity and development of chronic pain sensation by inducing maladaptive neuronal plasticity.

We found that SNI could induce mechanical allodynia and thermal hyperalgesia in mice. However, other studies in rats reported finding no detectable thermal hyperalgesia related to SNI (Decosterd and Woolf, 2000; Yamamoto et al., 2013; Zhou et al., 2017; Roth et al., 2023), suggesting a possible difference among species. Moreover, the pain induced by the SNI model is very intense, and different degrees of ligation and surgical (nerve) damage may result in different levels of thermal sensitivity in mice. When severe nerve injury leads to high intensity pain, pain signal transduction is not sensitive, which could potentially obscure thermal hyperalgesia. For example, some studies have reported that no thermal reflex is detected in the early stages after surgery, such as SNI 1D, whereas the induction of thermal hyperalgesia can be observed at SNI 3D or longer after surgery (Guida et al., 2015). Additionally, the temperature and duration of the thermal stimulus could also affect the results of thermal sensitivity. However, in other studies, the SNI has been shown to induce thermal hyperalgesia (Abe et al., 2011; Karl et al., 2017; Wang et al., 2017; Borgonetti and Galeotti, 2022; Borgonetti et al., 2022; Jin et al., 2022; Zhou et al., 2022). In addition, the threshold for thermal stimulation is lowered in SNI mice, resulting in pain sensitivity at lower temperatures (Abe et al., 2011; Jin et al., 2022; Zhou et al., 2022).

Chronic pain patients commonly report cognitive difficulties in clinical studies (Wiech et al., 2008; Moriarty et al., 2011; Bushnell et al., 2013; Wiech, 2016; Phelps et al., 2021). Chronic pain can also impair cognitive flexibility and memory in animal models (Liu and Chen, 2014; Cowen et al., 2018; Phelps et al., 2021), whereas improving cognitive behavior can reportedly serve as a treatment strategy for chronic pain by applying cognitive psychology techniques (Wiech, 2016; Phelps et al., 2021). Synaptic plasticity results in long-term negative effects, including persistent pain and cognitive impairment (Liu and Chen, 2014; Bliss et al., 2016). In chronic pain processing, changes in neuronal plasticity are associated with changes in cognitive function (Burke and Barnes, 2006; Erickson and Kramer, 2009; Ebbesson and Braithwaite, 2012). LCN2 has been linked to cognitive impairment (Dekens et al., 2021; Olson et al., 2021), and increased LCN2 levels have been observed in patients and mouse model of AD (Kang et al., 2021), as well as in the postmortem brain of patients with mild cognitive impairment (Choi et al., 2011). It is therefore reasonable to speculate that LCN2 could be involved in interactions between pain and cognition processes by regulating neuronal plasticity.

## Conclusion

In conclusion, the current study provides a theoretical basis for understanding the molecular mechanisms of neuronal plasticity in chronic pain. More specifically, our findings illustrate the functional role of LCN2 in inducing ACC^Glu^ neuronal hyperactivity in the development and maintenance of chronic pain, suggesting that LCN2 might serve as viable therapeutic target for treating chronic pain.

## Data availability statement

The original contributions presented in this study are included in the article/Supplementary material, further inquiries can be directed to the corresponding authors.

## Ethics statement

This animal study was reviewed and approved by the Animal Care and Use Committee of the University of Science and Technology of China.

## Author contributions

WW, WT, and ZZ: conceptualization and funding acquisition. X-JS, C-LY, PC, and AJ: methodology. X-JS, C-LY, YY, and YM: software. X-JS, C-LY, YY, YM, PC, and AJ: formal analysis. X-JS, PC, and AJ: investigation. X-JS: writing—original draft. X-JS and ZZ: writing—review and editing. X-JS, WW, WT, and ZZ: supervision. All authors contributed to the article and approved the submitted version.

## Supplementary material

Supplementary Video 1 *In vivo* two-photon calcium imaging of ACC^Glu^ neurons in sham and SNI 2W mice. Scale bar, 100 μm.
