# Supplementary material for: Up-regulation of LCN2 in the anterior cingulate cortex contributes to neural injury-induced chronic pain
Source: Front Cell Neurosci. 2023 Jun 8;17:1140769. doi: 10.3389/fncel.2023.1140769 (PMC10285483; doi:10.3389/fncel.2023.1140769)
Supplement: Supplementary file 1 [file Table_1.docx]

| Figure | Statistical method | Conditions | *t* or *F* value | *P* value | Number of samples |
| --- | --- | --- | --- | --- | --- |
| Fig. 1B | Two-way ANOVA | Withdrawal threshold | F (1, 70) = 249.3 | <0.0001 |  |
|  |  | BL: Sham vs. SNI |  | >0.9999 | n=8 mice per group |
|  |  | 3D: Sham vs. SNI |  | <0.0001 | n=8 mice per group |
|  |  | 7D: Sham vs. SNI |  | <0.0001 | n=8 mice per group |
|  |  | 10D: Sham vs. SNI |  | <0.0001 | n=8 mice per group |
|  |  | 14D: Sham vs. SNI |  | <0.0001 | n=8 mice per group |
| Fig. 1C | Two-way ANOVA | Latency | F (1, 70) = 34.81 | <0.0001 |  |
|  |  | BL: Sham vs. SNI |  | >0.9999 | n=8 mice per group |
|  |  | 3D: Sham vs. SNI |  | 0.0211 | n=8 mice per group |
|  |  | 7D: Sham vs. SNI |  | 0.0085 | n=8 mice per group |
|  |  | 10D: Sham vs. SNI |  | 0.0052 | n=8 mice per group |
|  |  | 14D: Sham vs. SNI |  | 0.0021 | n=8 mice per group |
| Fig. 2B | Two-tailed unpaired *t*-test | c-Fos cells/mm^2^ |  |  |  |
|  |  | Sham vs. SNI 2W | t_14_=11.47 | <0.0001 | n=8 slices per group |
| Fig. 2G | Two-way ANOVA | Firing Rate | F (1, 38) = 58.98 | <0.0001 |  |
|  |  | Sham vs. SNI 2W |  | <0.0001 | n=20 cells per group |
| Fig. 2H | Two-tailed unpaired *t*-test | Rheobase (pA) |  |  |  |
|  |  | Sham vs. SNI 2W | t_38_=5.781 | <0.0001 | n=20 cells per group |
| Fig. 2L | Mann-Whitney U test | *ΔF/F* (%) |  |  |  |
|  |  | Sham vs. SNI 2W | *U* = 2424 | 0.0079 | n=80 cells per group |
| Fig. 3E | Two-way ANOVA | Withdrawal threshold | F (1, 112) = 116.3 | <0.0001 |  |
|  |  | BL: SNI + mCherry vs. SNI + hM4Di |  | >0.9999 | n=8 mice per group |
|  |  | 14D: SNI + mCherry vs. SNI + hM4Di |  | 0.9999 | n=8 mice per group |
|  |  | 0.5h: SNI + mCherry vs. SNI + hM4Di |  | 0.971 | n=8 mice per group |
|  |  | 1.0h: SNI + mCherry vs. SNI + hM4Di |  | 0.0013 | n=8 mice per group |
|  |  | 1.5h: SNI + mCherry vs. SNI + hM4Di |  | <0.0001 | n=8 mice per group |
|  |  | 2.0h: SNI + mCherry vs. SNI + hM4Di |  | <0.0001 | n=8 mice per group |
|  |  | 3.0h: SNI + mCherry vs. SNI + hM4Di |  | 0.0017 | n=8 mice per group |
|  |  | 4.0h: SNI + mCherry vs. SNI + hM4Di |  | 0.512 | n=8 mice per group |
| Fig. 3F | Two-way ANOVA | Latency | F (1, 112) = 28.56 | <0.0001 |  |
|  |  | BL: SNI + mCherry vs. SNI + hM4Di |  | >0.9999 | n=8 mice per group |
|  |  | 14D: SNI + mCherry vs. SNI + hM4Di |  | >0.9999 | n=8 mice per group |
|  |  | 0.5h: SNI + mCherry vs. SNI + hM4Di |  | 0.9109 | n=8 mice per group |
|  |  | 1.0h: SNI + mCherry vs. SNI + hM4Di |  | 0.0091 | n=8 mice per group |
|  |  | 1.5h: SNI + mCherry vs. SNI + hM4Di |  | 0.0032 | n=8 mice per group |
|  |  | 2.0h: SNI + mCherry vs. SNI + hM4Di |  | 0.0032 | n=8 mice per group |
|  |  | 3.0h: SNI + mCherry vs. SNI + hM4Di |  | 0.0251 | n=8 mice per group |
|  |  | 4.0h: SNI + mCherry vs. SNI + hM4Di |  | 0.9906 | n=8 mice per group |
| Fig. 3K | Two-way ANOVA | Withdrawal threshold | F (1, 98) = 53.69 | <0.0001 |  |
|  |  | BL: Control + mCherry vs. Control + hM3Dq |  | >0.9999 | n=8 mice per group |
|  |  | 0.5h: Control + mCherry vs. Control + hM3Dq |  | 0.741 | n=8 mice per group |
|  |  | 1.0h: Control + mCherry vs. Control + hM3Dq |  | 0.0029 | n=8 mice per group |
|  |  | 1.5h: Control + mCherry vs. Control + hM3Dq |  | <0.0001 | n=8 mice per group |
|  |  | 2.0h: Control + mCherry vs. Control + hM3Dq |  | <0.0001 | n=8 mice per group |
|  |  | 3.0h: Control + mCherry vs. Control + hM3Dq |  | 0.0176 | n=8 mice per group |
|  |  | 4.0h: Control + mCherry vs. Control + hM3Dq |  | 0.9615 | n=8 mice per group |
| Fig. 3L | Two-way ANOVA | Latency | F (1, 98) = 39.66 | <0.0001 |  |
|  |  | BL: Control + mCherry vs. Control + hM3Dq |  | >0.9999 | n=8 mice per group |
|  |  | 0.5h: Control + mCherry vs. Control + hM3Dq |  | 0.2246 | n=8 mice per group |
|  |  | 1.0h: Control + mCherry vs. Control + hM3Dq |  | 0.0328 | n=8 mice per group |
|  |  | 1.5h: Control + mCherry vs. Control + hM3Dq |  | 0.0014 | n=8 mice per group |
|  |  | 2.0h: Control + mCherry vs. Control + hM3Dq |  | 0.0068 | n=8 mice per group |
|  |  | 3.0h: Control + mCherry vs. Control + hM3Dq |  | 0.0223 | n=8 mice per group |
|  |  | 4.0h: Control + mCherry vs. Control + hM3Dq |  | 0.5819 | n=8 mice per group |
| Fig. 4A | Two-tailed unpaired *t*-test | Relative LCN2 protein expression |  |  |  |
|  |  | Sham vs. SNI 2W | t_10_=3.291 | 0.0081 | n=6 mice per group |
| Fig. 4C | Two-tailed unpaired *t*-test | Normalized LCN2 level |  |  |  |
|  |  | Sham vs. SNI 2W | t_14_=5.872 | <0.0001 | n=8 slices per group |
| Fig. 4D | Two-tailed unpaired *t*-test | Relative LCN2 protein expression |  |  |  |
|  |  | Sham 3D vs. SNI 3D | t_10_=3.588 | 0.0049 | n=6 mice per group |
| Fig. 4F | Two-tailed unpaired *t*-test | Normalized LCN2 level |  |  |  |
|  |  | Sham 3D vs. SNI 3D | t_14_=6.993 | <0.0001 | n=8 slices per group |
| Fig. 5D | Two-tailed unpaired *t*-test | Co-labelled area |  |  |  |
|  |  | Sham vs. SNI 2W | t_10_=7.414 | <0.0001 | n=6 mice per group |
| Fig. 5F | Two-tailed unpaired *t*-test | Co-labelled area |  |  |  |
|  |  | Sham vs. SNI 2W | t_10_=0.0171 | 0.9867 | n=6 mice per group |
| Fig. 5H | Two-tailed unpaired *t*-test | Co-labelled area |  |  |  |
|  |  | Sham vs. SNI 2W | t_10_=0.5748 | 0.5781 | n=6 mice per group |
| Fig. 5L | Two-tailed unpaired *t*-test | Normalized 24p3R level |  |  |  |
|  |  | Sham vs. SNI 2W | t_14_=1.449 | 0.1694 | n=8 slices per group |
| Fig. 6D | Two-tailed unpaired *t*-test | Relative LCN2 protein expression |  |  |  |
|  |  | EGFP vs. shLCN2 | t_10_=5.805 | 0.0002 | n=6 mice per group |
| Fig. 6E | Two-way ANOVA | Withdrawal threshold | F (1, 28) = 6.962 | 0.0134 |  |
|  |  | Sham: EGFP vs. shLCN2 |  | >0.9999 | n=8 mice per group |
|  |  | SNI: EGFP vs. shLCN2 |  | 0.0005 | n=8 mice per group |
| Fig. 6F | Two-way ANOVA | Latency | F (1, 28) = 4.569 | 0.0414 |  |
|  |  | Sham: EGFP vs. shLCN2 |  | >0.9999 | n=8 mice per group |
|  |  | SNI: EGFP vs. shLCN2 |  | 0.0027 | n=8 mice per group |
| Fig. 6I | Two-way ANOVA | Firing Rate | F (1, 34) = 34.58 | <0.0001 |  |
|  |  | SNI + EGFP vs. SNI + shLCN2 |  | <0.0001 | n=18 cells per group |
| Fig. 6J | Two-tailed unpaired *t*-test | Rheobase (pA) |  |  |  |
|  |  | SNI + EGFP vs. SNI + shLCN2 | t_34_=4.751 | <0.0001 | n=18 cells per group |
| Fig. 7B | Two-way ANOVA | Withdrawal threshold | F (1, 28) = 12.45 | 0.0015 |  |
|  |  | Sham: Isotype mAb vs. LCN2 mAb |  | >0.9999 | n=8 mice per group |
|  |  | SNI: Isotype mAb vs. LCN2 mAb |  | <0.0001 | n=8 mice per group |
| Fig. 7C | Two-way ANOVA | Latency | F (1, 28) = 6.400 | 0.0173 |  |
|  |  | Sham: Isotype mAb vs. LCN2 mAb |  | >0.9999 | n=8 mice per group |
|  |  | SNI: Isotype mAb vs. LCN2 mAb |  | 0.0020 | n=8 mice per group |
| Fig. 7E | Two-way ANOVA | Firing Rate | F (1, 38) = 50.23 | <0.0001 |  |
|  |  | SNI + Isotype mAb vs. SNI + LCN2 mAb |  | <0.0001 | n=20 cells per group |
| Fig. 7F | Mann-Whitney *U* test | Rheobase (pA) |  |  |  |
|  |  | SNI + Isotype mAb vs. SNI + LCN2 mAb | *U* = 50 | <0.0001 | n=20 cells per group |
| Fig. 7H | One-way ANOVA | Inflammation scores | F (2, 15) = 5 | 0.0123 |  |
|  |  | SNI + ACSF vs. SNI + Isotype mAb |  | >0.9999 | n=6 mice per group |
|  |  | SNI + ACSF vs. SNI + LCN2 mAb |  | 0.0196 | n=6 mice per group |
|  |  | SNI + Isotype mAb vs. SNI + LCN2 mAb |  | 0.0400 | n=6 mice per group |
| Fig. 8B | Two-way ANOVA | Withdrawal threshold | F (1, 28) = 21.48 | <0.0001 |  |
|  |  | Pre: ACSF vs. rmLCN2 |  | >0.9999 | n=8 mice per group |
|  |  | Post: ACSF vs. rmLCN2 |  | <0.0001 | n=8 mice per group |
| Fig. 8C | Two-way ANOVA | Latency | F (1, 28) = 6.140 | 0.0195 |  |
|  |  | Pre: ACSF vs. rmLCN2 |  | >0.9999 | n=8 mice per group |
|  |  | Post: ACSF vs. rmLCN2 |  | 0.0014 | n=8 mice per group |
| Fig. 8E | Two-way ANOVA | Firing Rate | F (1, 38) = 36.60 | <0.0001 |  |
|  |  | ACSF vs. rmLCN2 |  | <0.0001 | n=20 cells per group |
| Fig. 8F | Two-tailed unpaired *t*-test | Rheobase (pA) |  |  |  |
|  |  | ACSF vs. rmLCN2 | t_38_=3.879 | <0.0001 | n=20 cells per group |
| Fig. 8G | Two-tailed unpaired *t*-test | V rest (mV) |  |  |  |
|  |  | ACSF vs. rmLCN2 | t_38_=2.671 | 0.0111 | n=20 cells per group |
| Fig. 8I | Two-tailed unpaired *t*-test | Input resistance (MΩ) |  |  |  |
|  |  | ACSF vs. rmLCN2 | t_38_=3.737 | 0.0006 | n=20 cells per group |
| Fig. 8L | Two-way ANOVA | Firing Rate | F (1, 38) = 31.89 | <0.0001 |  |
|  |  | ACSF vs. rmLCN2 |  | <0.0001 | n=20 cells per group |
| Fig. 8M | Two-tailed unpaired *t*-test | Rheobase (pA) |  |  |  |
|  |  | ACSF vs. rmLCN2 | t_38_=4.832 | <0.0001 | n=20 cells per group |
| Fig. 8N | Two-tailed unpaired *t*-test | V rest (mV) |  |  |  |
|  |  | ACSF vs. rmLCN2 | t_38_=2.672 | 0.0110 | n=20 cells per group |
| Fig. 8P | Two-tailed unpaired *t*-test | Input resistance (MΩ) |  |  |  |
|  |  | ACSF vs. rmLCN2 | t_38_=2.777 | 0.0085 | n=20 cells per group |
