# Supplementary material for: Up-regulation of LCN2 in the anterior cingulate cortex contributes to neural injury-induced chronic pain
Source: Front Cell Neurosci. 2023 Jun 8;17:1140769. doi: 10.3389/fncel.2023.1140769 (PMC10285483; doi:10.3389/fncel.2023.1140769)
Supplement: Supplementary file 2 [file Table_2.docx]

| **Resource or Reagent** | **Source** | **Identifier** |
| --- | --- | --- |
| **Antibodies** |  |  |
| Rabbit anti-c-Fos | Synaptic Systems | Cat# 226008 |
| Goat anti-LCN2 | BOSTER | Cat# PB9609 |
| Rabbit anti-LCN2 | Proteintech | Cat# 26991-1-AP |
| Rabbit anti-24p3R | Sigma-Aldrich | Cat# SAB3500306 |
| Mouse anti-NeuN | Millipore | Cat# MAB377 |
| Rabbit anti-Iba1 | Wako | Cat# 019-19741 |
| Rabbit anti-GFAP | Dako | Cat# Z0334 |
| Rabbit anti-glutamate | Sigma-Aldrich | Cat# G6642 |
| Mouse anti-glutamate | Sigma-Aldrich | Cat# G9282 |
| Mouse anti-GABA | Sigma-Aldrich | Cat# A0310 |
| Alexa fluor 488-anti-Goat secondary antibody | Invitrogen | Cat# A11055 |
| Alexa fluor 488-anti-Mouse secondary antibody | Invitrogen | Cat# A21202 |
| Alexa fluor 594-anti-Mouse secondary antibody | Invitrogen | Cat# A21203 |
| Alexa fluor 594-anti-Rabbit secondary antibody | Invitrogen | Cat# A21207 |
| Alexa fluor 594-anti-Goat secondary antibody | Invitrogen | Cat# A11058 |
| Alexa fluor 488-anti-Rabbit secondary antibody | Invitrogen | Cat# A21206 |
| β-actin | Absin | Cat# abs137975 |
| HRP Goat Anti-Mouse lgG (H+L) | Beyotime | Cat# A0216 |
| HRP Goat Anti-Rabbit lgG (H+L) | ABclonal | Cat# AS014 |
| **Bacterial and virus strains** |  |  |
| rAAV-CaMKIIa-GCaMP6f-WPRE-pA | BrainVTA | Cat# PT-0119 |
| rAAV-Ef1α-DIO-hM3D(Gq)-mCherry-WPRE-pA | BrainVTA | Cat# PT-0042 |
| rAAV-Ef1α-DIO-hM4D(Gi)-mCherry-WPRE-pA | BrainVTA | Cat# PT-0043 |
| rAAV-Ef1α-DIO-mCherry-WPRE-pA | BrainVTA | Cat# PT-0013 |
| rAAV9-U6-shRNA(LCN2)-CMV-EGFP | BrainCase | N/A |
| rAAV9-U6-shRNA(Scramble)-CMV-EGFP | BrainCase | N/A |
| **Chemicals** |  |  |
| LCN2 mAb | R&D Systems | Cat# MAB1857 |
| Isotype mAb | R&D Systems | Cat# MAB006 |
| Recombinant Mouse Lipocalin-2 (rmLCN2) | R&D Systems | Cat# 1857-LC |
| Clozapine-N-oxide (CNO) | MCE | Cat# HY-17366 |
